# The Immunomodulatory Effects of A2 β-Casein on Immunosuppressed Mice by Regulating Immune Responses and the Gut Microbiota

**DOI:** 10.3390/nu16040519

**Published:** 2024-02-13

**Authors:** Xiao Li, Xingru Lu, Ming Liu, Yu Zhang, Yujun Jiang, Xinyan Yang, Chaoxin Man

**Affiliations:** Key Laboratory of Dairy Science, Ministry of Education, College of Food Science, Northeast Agricultural University, Harbin 150030, China; lx17884103166@163.com (X.L.); xingru_lu0922@163.com (X.L.); lm2001100218@163.com (M.L.); jessedevil@163.com (Y.Z.); yujun_jiang@163.com (Y.J.)

**Keywords:** A2 β-casein, protein digestives, immune cell, immunoregulation, gut microbiota

## Abstract

The aim of this study was to investigate the immunomodulatory effects of A2 β-casein (β-CN) in cyclophosphamide-induced immunosuppressed BALB/c mice. Experiments conducted in vitro revealed that A2 β-CN digestive products have potent immunostimulatory activities. Animal studies demonstrated that A2 β-CN improved the immunological organ index reduction trend caused by cyclophosphamide, reduced the pathological damage to the spleen tissue in immunosuppressed mice, increased the release of IL-17A, IgG, and IgA, and reduced the production of IL-4. By regulating the relative abundance of advantageous bacteria like *Oscillospira*, *Lactobacillus*, and *Bifidobacteria* and harmful bacteria like *Coprococcus* and *Desulfovibrionaceae*, A2 β-CN improved gut microbiota disorders in immunosuppressed mice. Moreover, A2 β-CN promoted the production of short-chain fatty acids and increased the diversity of the gut microbiota. Therefore, ingestion of A2 β-CN is beneficial to the host’s immune system and gut health. These findings provide insights for the future application of A2 β-CN-related dairy products.

## 1. Introduction

Protein is the main ingredient in milk, and 80% of it is CN [1]. Based on the homology of the AA sequences, there are four different types of CN: αs1-CN, αs2-CN, β-CN, and κ-CN [2]. Among them, β-CN is the main protein in the CN fraction, accounting for about 30–40%. There are 13 genetic variants of β-CN: A1, A2, A3, B, C, D, E, F, G, H1, H2, I, and J [3]. A1 and A2 are the main variants, differing only by the 67th AA, which is His in A1 and Pro in A2 [4]. Structural differences between variants can impact milk properties and bioactivity. According to the literature [5], A2 β-CN is less hydrophobic and its dairy products have a tighter micellar structure, and A2 β-CN also shows higher molecular chaperone activity against heat-induced whey protein aggregation during heat treatment than A1 β-CN. Therefore, dairy products containing A2 β-CN have a better stability than those containing A1 β-CN [6]. Other studies showed that the His67 in A1 β-CN confers reduced sensitivity to proteolytic cleavage by digestive enzymes, leading to the release of β-casomorphin-7 [7,8]. Numerous gastrointestinal conditions, including stomach pain and changes in stool consistency, have been linked to β-casomorphin-7 [9,10], and may cause other health conditions such as immune disorders [11,12]. Ingestion of milk or dairy products containing A2 β-CN results in little or no intestinal release of β-casomorphin-7 [13].

In most studies on A1 and A2 β-CN, animal models have been employed to compare their functional properties. For example, one study [9] found that an A2 β-CN milk-based diet accelerated gastrointestinal motility in rats in contrast to A1 β-CN, which showed the opposite trend and induced localized inflammation. Another study [14] found similar results and showed that consumption of A1 variants (A1A1 and A1A2) induced an intestinal inflammatory response through activation of the T helper 2 pathway, whereas no changes occurred with consumption of A2 β-CN variants (A2A2). Additionally, another study [15] reported that consuming A2 milk lowered cholesterol levels and prevented ischemic heart disease.

In summary, although studies have shown that the physicochemical properties of A2 β-CN milk are more stable and more beneficial to human gastrointestinal health than A1 β-CN milk, the possible relationship between the digestive properties of A2 β-CN and human health needs to be further explored in preclinical tests, which allow us to gather more direct evidence and establish functional tests to evaluate the in vivo immunomodulatory aspects of A2 β-CN. Therefore, the present study aimed to evaluate the immunoreactivity of A2 β-CN digested both in vitro and in cyclophosphamide (CTX)-induced immunosuppressed mice in vivo. The immune organs, immune cells, serum biochemical indexes, immunoglobulins, and other indexes of A2 β-CN-containing dietary intervention were measured, and the changes in the structure of intestinal flora and short-chain fatty acids (SCFAs) in the cecum contents of mice were detected via 16S RNA and gas chromatography (GC) in order to assess the immune-modulating ability of A2 β-CN in immunosuppressed mice. This information will provide a theoretical basis and reference value for other functional foods.

## 2. Materials and Methods

### 2.1. Milk Samples

A2 milk was obtained from purebred A2 genotype cows located on a farm in northeast China. A1 milk was purchased from shopping malls and supermarkets in northeast China.

### 2.2. Extraction of A2 β-CN

Milk samples of 100 mL were taken, homogenized for 1 min, and then centrifuged at 5000× *g* for 20 min at 4 °C to remove most of the fat. The pH was increased to 4.6–4.7. After leaving the solution to stand for 30 min, β-CN precipitation could be observed. Precipitated CN was neutralized by washing with distilled water and it was transferred to dialysis bags. For 48 h, the dialysis bags were submerged in deionized water at 4 °C; the dialysate was replaced every 6 to 8 h. After dialysis, the protein solution was freeze-dried and stored at −20 °C. The β-CN in the crude extracted casein was separated and purified using a DEAE Bestarose Fast Flow (Bio-Rad, Hercules, CA, USA)) anion-exchange column using a GE AKTA Pure protein purifier (Bio-Rad, Hercules, CA, USA). Sodium dodecyl sulfate polyacrylamide gel electrophoresis (SDS-PAGE) was used to detect and identify the protein purification solution with the aim of identifying the type and purity of the protein. It was then desalted, and a BCA protein concentration kit was used to measure the protein content.

### 2.3. Preparation of Digestion Products

β-CN digests were prepared using a static in vitro digestion system according to the method in [16] with some modifications. A 4% A2 β-CN solution was used, and saliva, gastric juice, and intestinal juice were prepared simultaneously. The specific operation methods of the test were as follows:

Oral phase: 4% β-CN solution was mixed with simulated saliva, and the pH value was adjusted to 6.8. The reaction system was placed in an incubator at 37 °C and 130 rpm for 10 min.

Gastric phase: 0.2 g Nacl and 0.7 mL Hcl were mixed, and the volume was fixed to 100 mL. The sample solution was mixed 1:1 with the simulated gastric fluid, and 0.064 g of pepsin (3000 U/mg) was added. The pH value was adjusted to 2.5 and the culture conditions were the same as above. Samples were removed at 0.5 h, 1.0 h, 1.5 h, and 2.0 h.

Intestinal phase: Cacl_2_ at 36.7 mg/mL and Nacl at 219.1 mg/mL were prepared. Then, 1.5 mL Cacl_2_ and Nacl were added, and 1.8 g bile salt was added. The pH value was adjusted to 7.0, and 1.2 g trypsin (10,000 U/mg) was added. The culture conditions were the same as above and samples were removed at 2.5 h, 3.0 h, 3.5 h, 4.0 h, and 5.0 h.

### 2.4. A2 β-CN Immune Cell Assay In Vitro

#### 2.4.1. Cells

We obtained mice lymphoma cells (YAC-1) and mononuclear macrophages (RAW 264.7) from Shanghai Biological Technology Co., Ltd. (EK, Shanghai, China).

#### 2.4.2. Determination of Splenic Lymphocyte Proliferation

Cells were aseptically extracted [17], and the cell concentration was finally adjusted to 2 × 10^7^ cells/mL. Aliquots of 100 μL of this spleen lymphocyte suspension were inoculated in 96-well plates, and 100 μL of β-CN digest was added to obtain final concentrations of 0, 25, 50, and 100 μg/mL. Concanavalin A (ConA) was added, along with RPMI-1640 (Aladdin, Shanghai, China ), as a blank control well, and cultured for 48 h. After adding the Cell Counting Kit-8 (Aladdin, Shanghai, China) solution, the cells were grown for 4 h. The optical density value was determined at a wavelength of 570 nm, and the proliferation index (PI) of splenic lymphocytes was determined according to Equation (1):

PI = (OD value of experimental group)/(OD value of control group × 100%) (1)

#### 2.4.3. Phagocytosis Experiments of Macrophages

In 96-well plates, RAW 264.7 cells were seeded at a density of 2 × 10^5^ cells/mL. After 4 h, the cell supernatant was drained, and diluted β-CN digest was added to achieve final concentrations of 0, 25, 50, and 100 μg/mL. After 48 h, the dye solution (Neutral Red) (Amresco, OH, USA) was added, and the cells were rinsed twice or three times with PBS. After adding the cell lysis solution, the absorbance value at 540 nm was measured two hours later.

#### 2.4.4. Determination of Natural Killer Cell Activity

The concentration of YAC-1 cells was set to 1 × 10^4^ cells/mL and the concentration of spleen cells was set to 2 × 10^7^ cells/mL [18]. Effector cells and target cells were added at an effector/target ratio of 50:1. A total of 50 μL spleen cells and 50 μL target cells were added into the test well group X, 50 μL spleen cells into the effector cell control well group Y, and 50 μL target cells into the target cell control well group Z; three parallel groups were set up. An amount of 50 μL A2 β-CN digest was added to obtain final concentrations of 0, 25, 50, and 100 μg/mL. The absorbance values were measured at 450 nm after continued incubation for 4 h. NK cell activity was calculated according to Equation (2):

NK cell activity = [Z − (X − Y)]/Z × 100%(2)

### 2.5. Regulatory Effect of A2 β-CN in Immunosuppressed Mice

#### 2.5.1. Animals

Specific pathogen-free (SPF) BALB/c mice (6–8 wk old, *n* = 64, BW 18–20 g; Vital River, Beijing, China) were chosen to harvest the primary splenic lymphocyte suspension. The mice were adaptively raised for 7 d starting on the day of purchase at a relative temperature of 21 ± 1 °C and a relative humidity of 55 ± 5%. Animal use and care guidelines and ethical standards for animal protection were closely followed during animal research. The Animal Management Rules of the Ministry of Health, People’s Republic of China (documentation number 55, 2001), were strictly followed when using the animals in our experiment. The Animal Ethics Committee of Northeast Agriculture University approved the study’s experimental protocol (approved number: NEAUEC 20230418).

#### 2.5.2. Establishment and Treatment of Immunosuppressed BALB/c Mice Model

During the experiment, all mice were regularly gavaged every day with ad libitum access to drinking water. The mice were randomly divided into six groups, with 10 mice per group (12 mice in the model group (M)): the normal group (NC), the M group (M), the low-dose A2 β-CN group (LA2), the medium-dose A2 β-CN group (MA2), the high-dose A2 β-CN group (HA2), and the high-dose A1 β-CN group (HA1). From the 8th to the 10th d, the NC group was fed with normal saline. Other groups were given 80 mg/kg CTX for 3 d, and from the 11th to 35th d, groups NC and M were given normal saline by gavage. The LA2, MA2, and HA2 groups were treated by gavage with different doses of A2 β-CN (300, 600, and 900 mg/kg BW), and the CN group was treated with the same dose of A1 β-CN as the HA2 group. The dose by gavage was chosen according to the standard [19]. The specific grouping and gavage methods were carried out as described in Table 1.

To ascertain whether the modeling was successful, two mice from the NC and M groups were randomly euthanized three days into the experiment. (1) Compared with the mice in the NC group, the BW of mice in group M decreased by 15% ± 5%. (2) The number of white blood cells, neutrophils, and lymphocytes in peripheral blood decreased by 50%. (3) The spleen and thymus were taken and weighed to calculate the organ coefficients. A statistically significant difference in all the above indicators indicated successful modeling. Mice were weighed weekly and recorded. After the last gavage for 24 h, the mice were weighed, and except for the mice that needed to undergo the immunity-related experiments on the same day, the remaining mice were sacrificed, and the samples were collected, put into sterile freezing tubes, frozen in liquid nitrogen immediately, and kept in a refrigerator at −80 °C for spare use.

#### 2.5.3. Determination of Immune Organ Index

Mice were sacrificed according to the method in [20]. After eyeball blood collection, the mice were sacrificed by cervical dislocation, and the spleen and thymus were dissected in a sterile environment. The spleen and thymus tissues of the mice were weighed, and the organ index was calculated using Equation (3):

Organ index (mg/g) = Organ weight (mg)/Mouse weight (g)(3)

#### 2.5.4. Histopathological Observation of Spleen

After the mice were sacrificed and the spleens were removed, the surface color and texture of the spleen were observed and judged by touching the surface of the spleen. Following this overall observation, hematoxylin–eosin staining was performed. Paraffin sections were deparaffinized in water in order to achieve this. Hematoxylin was then used to stain the nuclei, and eosin was used to stain the cytoplasm. Lastly, the histopathological morphology and seal dewatering were noted.

#### 2.5.5. Spleen Lymphocyte PI

Spleens were harvested aseptically (see Section 2.4.2), and the cell concentration was 3 × 10^6^ cells/mL. Two wells of a 24-well culture plate were filled with the spleen cell suspension; one well received ConA solution (7.5 μg/mL) and the other well served as a control and was cultured for 3 d. The supernatant from each well was discarded 4 h prior to the completion of the culture. Next, RPMI-1640 medium without calf serum was added, followed by 3-(4,5-dimethylthiazol-2-yl)-2,5-diphenyltetrazolium bromide (5 mg/mL). Dimethyl sulfoxide was added and mixed, and aliquots were loaded into 96-well culture plates; three parallel wells were repeated. Absorbance values were measured at 570 nm, and the lymphocyte stimulation index was calculated using Equation (4):

Lymphocyte stimulation index = (OD of experimental group − OD of blank group)/(OD of control group − OD of blank control group)(4)

#### 2.5.6. Phagocytosis Index and Percentage of Peritoneal Macrophages

Before the immunization test, the mice were i.p. injected with 0.2 mL of 4% sheep red blood cells (RBCs). Mice were sacrificed on the day of the test and injected with Hank’s solution supplemented with calf serum. The abdominal wash was sucked into the test tube along the abdomen, and 0.5 mL was added to the test tube with the same volume of chicken RBC suspension. Then, 0.5 mL of the mixed liquid was sucked into the agar circle on the cover glass, incubated at 37 °C for approximately 15 min to dry, and then rinsed to remove the cells that did not adhere to the wall, fixed with propanol/methanol solution, and stained. The time of staining was determined according to the actual situation. After completion, the specimens were rinsed with distilled water, dried, and observed using a microscope at 100× magnification for counting. The phagocytosis rate (%) and phagocytic index were calculated according to Equations (5) and (6), respectively:

Percentage of phagocytosis (%) = ((Phagocytes engulfing chicken RBC))/((Number of 100 phagocytes)) ×100%(5)

Phagocytic index = (Number of phagocytosed chicken RBC)/(Number of 100 phagocytes)(6)

#### 2.5.7. Determination of NK Cell Activity

Following the procedure outlined in Section 2.4.2, a splenocyte suspension was created, and the cell concentration was increased to 2 × 10^7^ cells/mL. Then, 100 μL of target cells (YAC-1 cells) and effector cells (splenocytes) were added to U-type 96-well culture plates. RPMI-1640 culture medium and target cells were added to the natural release well, and 2.5% Triton and target cells were added to the maximum release well. After being aspirated, the supernatant was combined with the LDH matrix solution. A 1 M HCl solution was added after 3–10 min. At 490 nm, absorbance values were measured, and Equation (7) was used to determine the NK cell activity:

NK cell activity (%) = (OD of reaction well − OD of natural release well)/((OD of maximum release well − OD of natural release well) × 100%)(7)

#### 2.5.8. Determination of Immune Cytokines and Immunoglobulins

An appropriate amount of spleen was weighed and blended with normal saline in a ratio of 1:9. The spleen was homogenized using a tissue grinder over an ice water bath. The contents of immune cytokines IL-4 and IL-17A were determined by an ELISA kit (Hengyuan biological, Shanghai, China) at 4 °C.

After eyeball blood sampling, serum samples were collected to detect IgG and IgA levels via the corresponding kits.

#### 2.5.9. Determination of Serum Biochemical Indicators

Blood samples were extracted from the mice’s eyes and centrifuged for 10 min at 2000× *g*. An automatic biochemical analyzer was used to measure 5 serum biochemical parameters.

#### 2.5.10. 16S rDNA Sequencing and Analysis

Each mouse had its feces sampled, and the samples were kept at −80 °C. An OMEGA Soil DNA Kit (M5636-02) (Omega Bio-Tek, Norcross, GA, USA) was utilized to extract DNA. Using agarose gel electrophoresis and a NanoDrop NC2000 spectrophotometer (Molecular Devices, San Jose, CA, USA), the amount and quality of isolated DNA were determined. Using the reverse primer 806R (GGACTACHVGGGTWTCTAAT) and forward primer 338F (ACTCCTACGGGAGGCAGCA), the V3–V4 region of the bacterial 16S rRNA genes was amplified by PCR. The process of thermal cycling involved 5 min of initial denaturation at 98 °C, 25 cycles of denaturation at 98 °C, 30 s of annealing at 5 °C, 45 s of extension at 72 °C, and finally 5 min of extension at 72 °C. PCR amplicons were measured via the Invitrogen Quant-iT PicoGreen dsDNA Assay Kit (Invitrogen, Waltham, MA, USA). Following equal pooling of amplicons, pair-end 2–250 bp sequencing was carried out on the Illumina platform with the 500 cycle NovaSeq 6000 SP Reagent Kit (Illumina, San Diego, CA, USA).

#### 2.5.11. Determination of SCFAs

GC was used to determine the levels of SCFAs in stool samples. A fecal sample of 0.1 g was weighed into a centrifuge tube containing a saturated NaCl solution of 0.5 mL, homogenized, acidified by adding H_2_SO_4_, and spun for 30 s. Anhydrous ether was added, and the vessel stirred for 30 s. SCFAs were extracted at 14,000 rpm/min for 15 min. Anhydrous sodium sulfate solution was added to the supernatant, and the supernatant was centrifuged under the above conditions, filtered with a filter membrane, and transferred to a gas flask for analysis and determination.

### 2.6. Data Analysis

SPSS 27.0.1 software was used to perform a one-way ANOVA. Other statistical analyses were performed using Excel 2018. Data visualization and plotting were achieved using PeakFit v4.12 and GraphPad Prism 8.02. Every experiment was repeated three times, and the results are expressed as means ± SD. *p* < 0.05 indicates a significant difference, and *p* < 0.01 is considered highly significant.

## 3. Results

### 3.1. Effects of A2 β-CN Digests on Immune Cells

**Figure 1:**
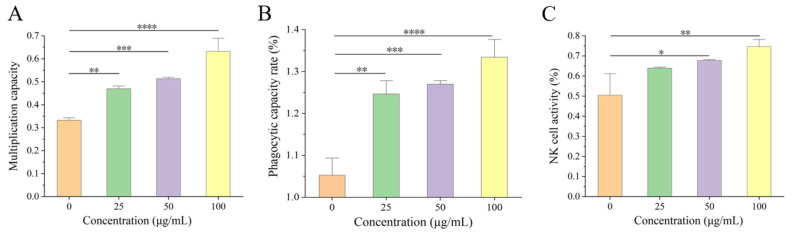
Effects of β-CN digests on three immune cells. (**A**) The proliferative capacity of splenic lymphocytes, (**B**) the phagocytic capacity of phagocytes, (**C**) NK cell activity. Comparison of different significance: * *p* < 0.05, ** *p* < 0.01, *** *p* < 0.001, **** *p* < 0.0001.

The effect of A2 β-CN digests on three types of immune cells was investigated using Cell Counting Kit-8, and the results are shown in Figure 1. Compared with the no A2 β-CN digest treatment, 25–100 μg/mL of the digest significantly increased the proliferation of splenic lymphocytes, the phagocytosis of macrophages, and NK cell activity. When treated with 100 μg/mL of the digest, the immune activity of the three kinds of cells increased by 90.07%, 26.72%, and 70.56% compared to the group without treatment.

### 3.2. Immunomodulatory Effects of A2 β-CN in Immunosuppressed Mice

#### 3.2.1. Effect of A2 β-CN on the Immune Organ Index

**Figure 2:**
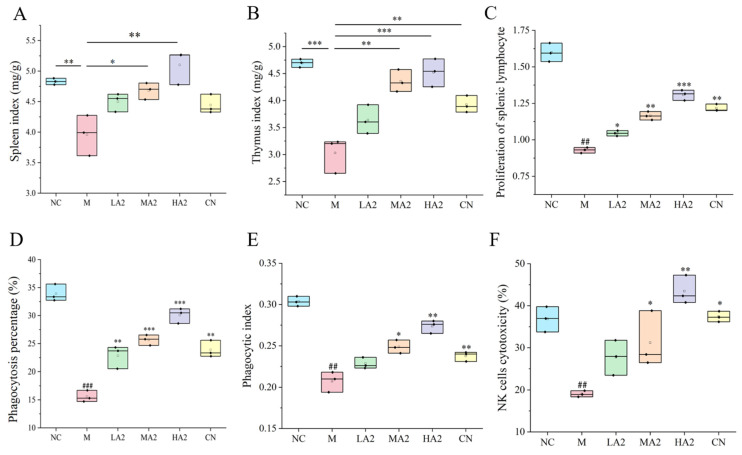
Effect of A2 β-CN on organ indexes and immune cells in mice. (**A**) Spleen index, (**B**) thymus index, (**C**) proliferation of splenic lymphocytes, (**D**) phagocytosis percentage, (**E**) phagocytic index, (**F**) NK cell activity. NC: control group, M: model group, LA2: low-dose A2 β-CN group, MA2: medium-dose A2 β-CN group, HA2: high-dose A2 β-CN group, CN: high-dose A1 β-CN group. Compared with the normal group:, ## *p* < 0.01, ### *p* < 0.001; compared with the model group: * *p* < 0.05, ** *p* < 0.01, *** *p* < 0.001.

The variations in the organ indexes of the mice in each group during the test course were studied (Figure 2A,B). The spleen/thymus index of the M-treated mice dramatically decreased (*p* < 0.01) in comparison to the NC group, indicating the successful establishment of the CTX model. Compared with M-treated mice, the spleen index was increased in all groups after gavage, especially in the MA2 and HA2 groups (*p* < 0.05 or *p* < 0.01). Compared with NC, both MA2 and HA2 were significantly higher than M (*p* < 0.01 or *p* < 0.001).

#### 3.2.2. Effect of A2 β-CN on the Activity of Immune Cells in Immunosuppressed Mice

ConA-induced proliferation of spleen lymphocytes in mice was shown (Figure 2C). The PI was significantly lower in group M than in group NC (*p* < 0.01). The PI was noticeably higher in the LA2, MA2, HA2, and CN groups than in group M and showed a dose-dependent relationship. Compared with group M, the percentage of total macrophages that were phagocytotic increased by 46.75% (*p* < 0.01), 64.87% (*p* < 0.001), and 93.28% (*p* < 0.001) in each gavage group, respectively, and the phagocytic index increased by 18.41% (*p* < 0.05) in group MA2 and 30.31% (*p* < 0.01) in group HA2 (Figure 2D,E). These results indicate that A2 β-CN could enhance the macrophages’ phagocytic function, thereby increasing the body’s nonspecific immunity.

Compared with group M, the NK cell activity of the mice treated with gavage of HA2 increased by 102.15% (*p* < 0.01) (Figure 2F). The results demonstrated that a high dose of A2 β-CN could boost NK cell activity and enhance the capacity of immune regulation.

#### 3.2.3. Influence of A2 β-CN on the Content of Immune Cytokines and Immunoglobulin

**Figure 3:**
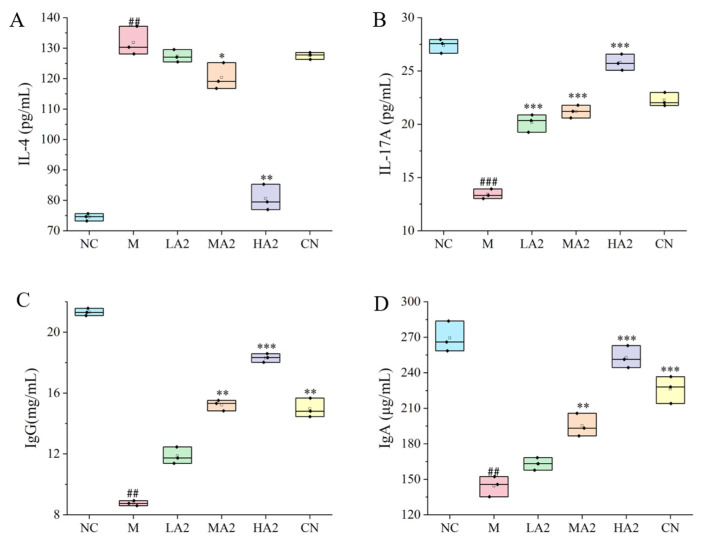
Effects of A2 β-CN on cytokines and immunoglobulins. (**A**) IL-4 content, (**B**) IL-17A content, (**C**) IgG content, (**D**) IgA content. Compared with the normal group: ## *p* < 0.01, ### *p* < 0.001; compared with the model group: * *p* < 0.05, ** *p* < 0.01, *** *p* < 0.001.

Group M’s serum levels of IL-17A, TNF-α, and IFN-γ were considerably lower (*p* < 0.001, *p* < 0.001, *p* < 0.01) than those of group NC, whereas IL-4 levels increased (*p* < 0.01). In contrast to group M, A2 β-CN intervention reduced the increase in IL-4 and, to variable degrees, slowed down the decrease in IL-17A brought on by CTX. Group CN did not significantly change (Figure 3A,B).

The CTX model was successfully established in M-treated mice, as evidenced by the decrease (*p* < 0.01) in IgG and IgA levels when compared to group NC. After gavage, the levels of IgG and IgA in each group were higher than in group M. The differences between groups MA2, HA2, and CN and group M were statistically significant (*p* < 0.01, *p* < 0.001, *p* < 0.001) (Figure 3C,D).

#### 3.2.4. Histopathological Observation of Spleens

**Figure 4:**
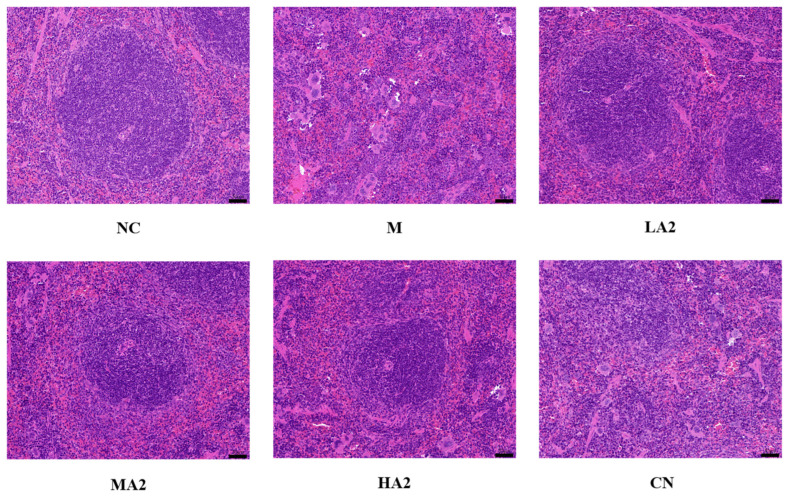
Effect of A2 β-casein on histopathology of spleen (H&E staining). Magnification: 200× and scale bar: 50 µm.

The morphological structure of splenic nodules in group M was severely scattered, presenting a disordered organizational structure with numerous multinucleated giant cells visible in the red pulp, and extramedullary hematopoietic cell clusters could be seen locally. In groups LA2 and MA2, the morphology and structure of splenic aggregates were normal, and the degree of splenic tissue injury was noticeably improved compared with group M. The tissue structure of group HA2 was essentially normal, and a few extramedullary hematopoietic cells were observed in the red marrow. These results indicated that a high dose of A2 β-CN can reduce the pathological damage of spleen tissue more effectively (Figure 4).

#### 3.2.5. Effects of A2 β-CN on Serum Biochemical Parameters in Immunosuppressed Mice

The results of the five serum biochemical indexes of the immunosuppressed mice are shown in Table 2. Compared with group NC, the values of all indexes in group M were decreased (*p* < 0.05), indicating the deleterious effect of CTX in mice. Compared with group M, the value of each index increased significantly after different doses of protein products (*p* < 0.05), tending to a healthy value, indicating that the β-CN products improved the physiological condition of mice.

For the four important biochemical indexes (ALB, ALT, TP, and BUN), compared with group M, the biochemical indexes of the protein intervention group increased by 9.69–30.34%, 12.34–44.75%, 28.34–53.32%, and 8.26–30.45%, respectively, compared with the same dose of HA2. The same four biochemical indexes for the CN group were decreased by 17.41%, 15.27%, 2.75%, and 5.68% compared to the HA2 group at the same dose.

#### 3.2.6. Effect of A2 β-CN on Gut Microbiota in Immunosuppressed Mice

To explore the effect of A2 β-CN on the gut microbiota in immunosuppressed mice, we analyzed the changes in gut microbial structure using 16S rRNA high-throughput sequencing.

**Figure 5:**
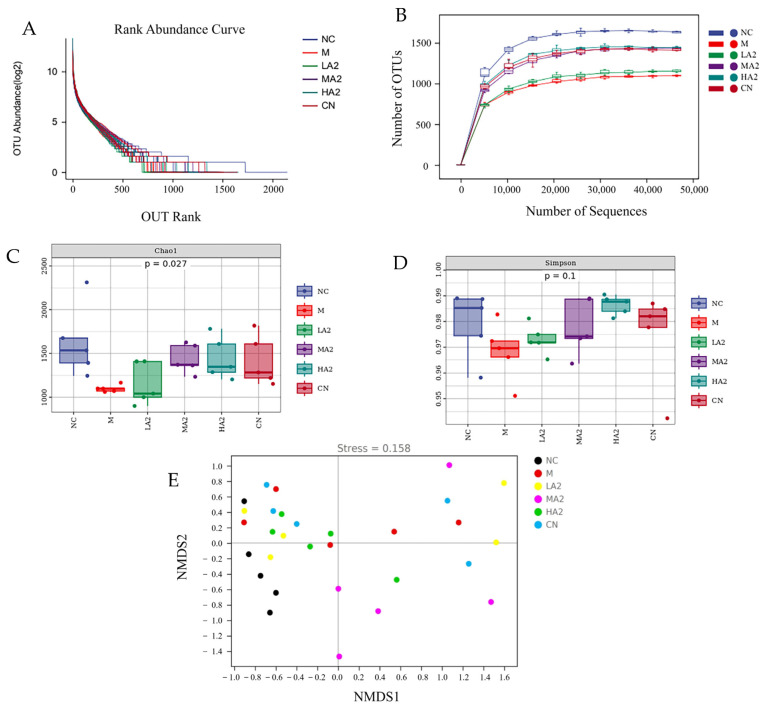
Effect of A2 β-CN on gut microbiota in immunosuppressed mice. (**A**) Dilution curve, (**B**) rank abundance curves, (**C**) Chao1 index, (**D**) Simpson index, (**E**) NMDS analysis.

The complexity and diversity of the sample species were analyzed using alpha diversity. With the increase in sequencing depth, the number of detected operational taxonomic units increased continuously, and the curves of six groups (NC, M, LA2, MA2, HA2, and CN) gradually increased, indicating that the sequencing depth covered most species and the richness of microbial composition was high (Figure 5). The rank abundance curve of gut microbiota in the six groups was relatively flat, and the species composition was relatively homogeneous (Figure 5A,B). Compared with group NC, the Chao1 index of group M decreased significantly and the Simpson index decreased, but there was no difference among the groups (Figure 5C,D). Compared with group M, the Simpson index of group HA2 was closer to that of group NC, which showed that treatment with HA2 could improve the gut microbiota diversity in CTX-induced immunosuppressed mice. These results indicate that the A2 β-CN diet can improve the richness and variety of gut microbiota in immunosuppressed mice.

Typically, beta diversity is employed to examine how species variety varies among samples. A non-metric multidimensional scaling (NMDS) analysis yields more dependable results when the stress value is less than 0.2. In the figure, each point denotes a single sample, and variously colored points stand for various sample groups. Since NMDS employs rank ordering, it can be roughly inferred that the difference in microbial communities between the two samples will be smaller (bigger) the closer (farther) the distance between two sites. In addition to reliably indicating the differences in colony composition between samples, the NMDS analysis stress value of 0.158 also allows for the distinction of various sample groups. The A2 β-CN diet, on the other hand, was shown to be helpful in stabilizing the structure of intestinal flora, as seen by the closer distance between the A2 β-CN dietary intervention group and the NC group (Figure 5E).

It can be seen that groups M and NC are essentially in separate quadrants, indicating that CTX affects the construction and composition of the microbiota. Under the A2 β-CN diet intervention, the distance between the A2 β-CN diet intervention group and the NC group was close, indicating that the A2 β-CN diet was conducive to stabilizing the gut microbiota structure.

**Figure 6:**
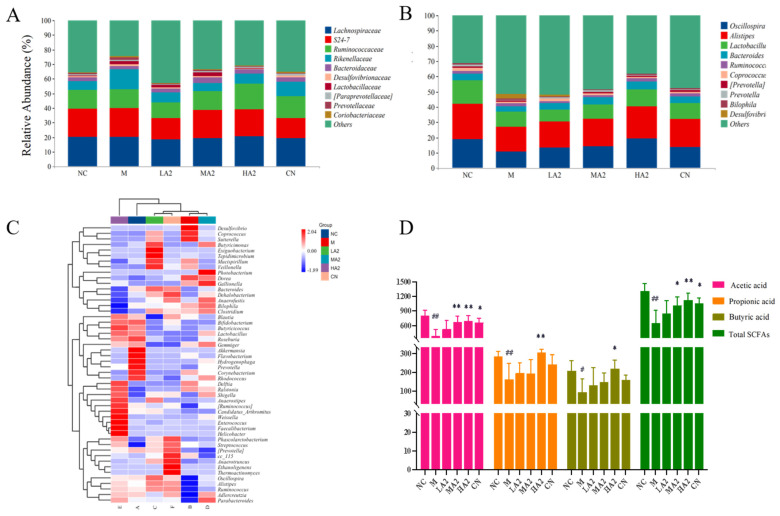
Effect of A2 β-CN on gut microbiota and SCFAs in immunosuppressed mice. (**A**) Department level, (**B**) genus level, (**C**) heat map of species composition at the genus level, (**D**) content of SCFAs. Compared with the normal group: # *p* < 0.05, ## *p* < 0.01; compared with the model group: * *p* < 0.05, ** *p* < 0.01.

To explore the effects of the A2 β-CN diet and CTX on the intestinal microbial communities in immunosuppressed mice, the microbial communities were classified at the department and genus levels, and the results are shown in Figure 6A. *Lachnospiraceae*, *S24-7*, and *Ruminococcaceae* were among the main families in each group. All three families were decreased in group M, but in the groups under the A2 β-CN diet intervention, they returned to normal levels. At the genus level, the relative abundance of *Oscillospira*, *Alistipes*, *Lactobacillus*, and *Bacteroides* in the gut microbiota of M-treated mice decreased, whereas the relative abundance of each of these four genera increased in all dose groups (Figure 6B). These results suggest that dietary intervention with A2 β-CN can improve or increase the relative abundance of Lactobacillus in mice and that this adjustment of the gut microbiota can alleviate dysbiosis in mice with CTX-induced immunosuppression.

A heatmap plot from the unweighted pair group method with arithmetic mean (UPGMA) cluster analysis was constructed based on the Euclidean distance of the species and sample composition data. CTX induction significantly affected normal mice and even altered colony abundance compared to the NC-treated mice and intestinal microecology was disrupted (Figure 6C). *Akkermansia*, *Bifidobacterium*, and *Butyricicoccus* were among the most abundant bacteria in group NC. The species composition of groups M and NC was significantly different at the genus level, with *Desulfovibrio*, *Coprococcus*, and *Sutterella*. Compared with group M, *Weissella*, *Ruminococcus*, and *Bifidobacterium* were among the dominant bacteria in group HA2. In summary, the gut microbiota structure of mice induced by CTX was more likely to colonize, including harmful bacteria, such as *Desulfovibrio* and *Coprococcus*. *Lactobacillus*, *Bifidobacterium*, and *Parabacteroides* were significantly increased in group HA2. The composition and abundance of gut microbiota in group HA2 were similar to those in group NC.

#### 3.2.7. Effect of A2 β-CN on Intestinal SCFA in Immunosuppressed Mice

We used GC to assess the acetic acid, propionic acid, butyric acid, and total acid levels in feces samples from immunosuppressed mice in order to confirm the effects of A2 β-CN on SCFAs generated by the microbiota. Acetic acid (*p* < 0.01), propionic acid (*p* < 0.01), and butyric acid (*p* < 0.05) were decreased in group M compared with group NC. Compared with group M, the acetic acid and total acid contents increased (*p* < 0.01 and *p* < 0.05) under MA2 dietary intervention. Furthermore, with the HA2 dietary intervention, there was an increase in the levels of acetic acid, propionic acid, butyric acid, and total acid (*p* < 0.05) (Figure 6D).

## 4. Discussion

As one of the body’s many defense mechanisms, the immune system plays a vital physiological role. By monitoring and recognizing self and non-self components, it protects the body by recognizing and eliciting an appropriate response to threats, such as toxins and invasion by pathogenic microorganisms, as well as removing internally damaged cells and maintaining body health [21]. The immune system of the spleen guards the body against pathogen invasion, so an analysis of the immune reactivity of spleen cells reveals the immune capacity of the host [22]. Proliferation of splenic lymphocytes is the most accurate measure of the immunological state of the host [23]. When infections are met, natural killer cells (NK cells) react promptly by secreting cytokines, including TNF-α and IFN-γ [24]. In addition, ingredients in food, including CN hydrolysate, whey protein and its enzymatic hydrolysate, and plant polysaccharides, have in vitro immune effects [25]. CN, a component with important nutritional value in food, plays a crucial role in immune regulation. On this basis, we found that β-CN digest dietary intervention at 25–100 μg/mL led to noticeably increased splenic lymphocyte proliferation, phagocytic capacity of macrophages, and NK cell activity in a dose-dependent pattern compared to no dietary intervention.

CTX is an alkylating agent that is used as an antineoplastic drug and an immunosuppressant [26]. It can cause significant immunosuppressive effects on the gastrointestinal tract, genital system, and immune system of animals and has been revealed to induce liver function damage and intestinal microbiota disorder and destroy intestinal mucosal immunity [27]. In the current study, CTX was used as an immunosuppressant to establish an immunosuppressive mice model, and the immune status of the mice was observed via examination of spleen tissue sections to determine the immunosuppressive effect.

Important immune system organs include the spleen and thymus, whose organ indices can provide insight into how well the body’s immune system is functioning [28]. Therefore, a decrease in the immune organ index is one of the representative symptoms of immune suppression in mice. We found that CTX induced immunosuppression in BALB/c mice, resulting in a decrease in the spleen index and thymus index in group M, while different doses of A2 β-CN noticeably augmented these two indexes and improved the growth and development of immunodeficient mice. The immunomodulatory effect of A1 β-CN was lower than that of A2 β-CN at the same dose. Compared with the NC group, the immunosuppressed mice showed a physiological state of low immune function. Hematoxylin–eosin staining indicated that the morphological structure of the spleen was severely scattered, the organizational structure was disordered, and the pathological damage was intense, which explained the decrease in the spleen index.

The main immune cells are NK cells, T cells, and macrophages. T cells are essential for cellular immunity [29]. The basis for the T lymphocyte proliferation experiment is the lymphocytes’ reactivity to mitogens. ConA has the ability to stimulate T cell transformation and proliferation, which modulates immunity. As a result, the capacity of spleen cells to proliferate when triggered by ConA may indicate how well the cells are functioning immunologically. The two main effector cells in innate immunity, macrophages and natural killer cells, are crucial in antitumor and anti-infection processes. In this study, we found significant reductions in the proliferation of splenic lymphocytes, phagocytosis of macrophages, and activity of NK cells, demonstrating that CTX noticeably inhibited the immune function of mice and destroyed the stability of the body’s immune system. However, different doses of A2 β-CN significantly increased the proliferation of splenic lymphocytes, the phagocytosis of macrophages, and NK cell activity, and the immune status was improved.

In the process of immunosuppression, the body will produce a series of inflammatory responses through the interaction of anti-inflammatory and pro-inflammatory factors. IL-4 is a multifunctional cytokine with an important role in cells and is involved in innate and acquired immunity in vivo [30]. Depending on the model of intestinal inflammation, it can play either a pro- or anti-inflammatory role as a key mediator of intestinal inflammation. One significant pro-inflammatory cytokine that has a role in the immune response is IL-17A. We found that the levels of pro-inflammatory cytokines in group M were observably lower than in group NC. Compared with group M, the serum level of IL-17A in the protein intervention group was significantly increased, and compared with group NC, the serum level of IL-17A in group M observably increased. In addition, the content of IL-4 in serum was significantly reduced after the gavage of medium and high doses of protein, which is consistent with other research results [31].

The serum biochemical indexes of experimental animals generally reflect the absorption and distribution of nutrients and their amount in the blood and further reflect the physiological state of experimental animals. Some studies have shown an increase in allergic or inflammatory responses after consumption of A1 milk compared with A2 milk. A study reported elevated levels of IgE and IgG in the serum of mice fed with A1 milk, and the intake of A1 β-CN compared with A2 β-CN induced an intestinal inflammatory response by activating the T helper 2 pathway [18]. However, another study found no difference in serum IgG levels between the groups of mice fed a diet containing lyophilized A1 milk and lyophilized A2 milk and the control group, and the level of intestinal inflammation was comparable among all three groups [32]. We found that our A2 β-CN dietary intervention could significantly increase the levels of IgG and IgA in the serum of mice, promote the production of proteins by lymphocytes in the immune system of mice, and induce the conversion of antigens to antibodies. Meanwhile, A2 β-CN showed a stronger immune-enhancing effect than A1 β-CN at the same dose.

With the development of gut microbiota research, diet is being increasingly considered as a key factor in the regulation of the gut microbiota. Many studies have reported the effect of proteins on the gut microbiota. For instance, they discovered that A2 β-CN altered the composition of the gut microbiota in aging mice [32]. Although A2 β-CN has attracted much attention, few studies have discussed the effect of A2 β-CN on intestinal health. In the current study, we explored the effects of A2 β-CN on intestinal health in immunosuppressed mice by measuring the alpha and beta diversity of the gut microbiota. At the family level, we found that an A2 β-CN diet significantly inhibited the colonization of harmful bacteria. At the genus level, we found that compared with the NC group, the relative abundance of the harmful bacteria *Coprococcus* and *Desulfovibrio* increased. Compared with group M, the A2 β-CN diet could increase the relative abundance of *Lactobacillus* and *Anobacterium* and reduce the relative abundance of *Coprococcus* and *Desulfovibrio* in mice. *Oscillospira* is widely distributed in the intestinal tract of animals and humans. At other levels, it has been shown to be associated with diseases such as chronic constipation, emaciation, and gallstones, as well as positive or negative changes in pathogenesis. *Alistipes*, a Gram-negative bacterium in the phylum *Bacteroidetes* and a relatively new genus, is found in the healthy human gut [33].

A heat map analysis of the species composition at the genus level showed that CTX induction observably affected the NC mice and even changed the colony abundance, and the intestinal microecology was destroyed. However, the A2 β-CN diet improved the CTX-induced flora imbalance to a certain extent, regulated the gut microbiota imbalance in immunosuppressed mice, and augmented the colonization of salutary bacteria in the gastrointestinal tract to make it closer to the level in NC mice and achieve the purpose of alleviating the reduced immune ability.

Acetic acid, propionic acid, butyric acid, valeric acid, caproic acid, and their isomers are examples of SCFAs, which are SFAs with a carbon chain consisting of one to six carbon atoms [34]. Their possible functions include immune system regulation, lipid metabolism, weight control, glucose homeostasis, and inflammatory responses [29,35]. It has been demonstrated that butyric and acetic acids control the body’s immunological response primarily by attaching to various receptors to encourage immune cell development and activate intestinal immune cells [36]. Moreover, it has been demonstrated that modifications to the gut microbiota directly impact the synthesis of SCFAs and quicken the host’s antibody responses [37].

In this study, we found that the content of SCFAs in the intestine of the group M mice was noticeably reduced, which is consistent with other research results [38]. It may be due to the reduced variety of gut microbiota in mice, as induced by CTX, resulting in a reduction in the species or the number of SCFA-producing microorganisms. On the other hand, the A2 β-CN diet has the potential to mitigate this decline, comparatively elevate the SCFA content, and markedly elevate the levels of acetic acid, propionic acid, butyric acid, and total acid.

## 5. Conclusions

The present study demonstrated that A2 β-CN can promote splenic lymphocyte proliferation and significantly increase the macrophage phagocytic index and NK cell activity. A2 β-CN can improve the concentration of SCFAs, adjust the diversity and composition of the gut microbiota in CTX-induced immunosuppressed BALB/c mice, enhance the intestinal mucosal immune function, including recovering the immune organ index, enhancing immune cell functions, and reducing spleen injury. The content of IL-17A in serum was significantly enhanced, the content of IL-4 cytokine was decreased, the contents of IgG and IgA in serum were significantly increased, the serum biochemical indicators tended to healthy values, and the physiological condition of mice was significantly improved. This result further suggests that A2 β-CN has the potential to act as a gut microbiota and immune modulator to reduce the harmful effects of CTX on the immune system. This research could offer an academic basis for the progress of A2 β-CN functional foods.

## Figures and Tables

**Table 1:** Groups of experimental mice and their daily treatments. (PBS: phosphate-buffered saline).

Group	Group Name	1–7 Days	8–10 Days	11–35 Days
Normal group	NC	Adaptive feeding	PBS	0.2 mL sterile PBS
Model group	M			0.2 mL sterile PBS
Low-dose A2 β-CN group	LA2			0.2 mL 300 mg/kg A2 β-CN
Medium-dose A2 β-CN group	MA2		80 mg/kg CTX	0.2 mL 600 mg/kg A2 β-CN
High-dose A2 β-CN group	HA2			0.2 mL 900 mg/kg A2 β-CN
High-dose A1 β-CN group	CN			0.2 mL 900 mg/kg A1 β-CN

**Table 2:** Effects of A2 β-CN on serum biochemical indices in immunosuppressed mice.

Biochemical Criterion	NC	M	LA2	MA2	HA2	CN
ALB (g/L)	24.58 ± 0.26 ^a^	18.30 ± 0.25 ^d^	20.07 ± 0.66 ^c^	21.13 ± 0.15 ^b^	23.85 ± 0.63 ^a^	19.70 ± 0.58 ^c^
ALT (U/L)	47.21 ± 2.05 ^a^	29.38 ± 1.88 ^e^	33.01 ± 0.16 ^d^	35.39 ± 0.96 ^cd^	42.52 ± 2.01 ^b^	36.03 ± 1.64 ^c^
TP (g/L)	47.87 ± 1.30 ^a^	32.93 ± 4.20 ^c^	42.26 ± 3.10 ^b^	46.95 ± 1.04 ^a^	50.49 ± 1.30 ^a^	49.10 ± 2.73 ^a^
BUN (mmol/L)	20.99 ± 0.51 ^a^	14.34 ± 0.92 ^c^	15.52 ± 1.62 ^c^	17.58 ± 1.28 ^b^	18.71 ± 0.96 ^b^	17.64 ± 0.25 ^b^
GLU (mmol/L)	5.08 ± 0.17 ^a^	1.71 ± 0.09 ^d^	2.99 ± 0.59 ^c^	3.34 ± 0.79 ^c^	4.43 ± 0.19 ^ab^	4.18 ± 0.17 ^b^

Note: Duncan’s multiple comparison analysis was used, with different letters in the same row representing significant differences (*p* < 0.05). (ALB: Albumin; ALT: Alanine aminotransferase; TP: Total protein; BUN: Blood urea nitrogen; GLU: Glutamic acid).

## Data Availability

The datasets used and analyzed in this study are available from the corresponding author on reasonable request.

## References

[1] Gomes-Santos A.C., Fonseca R.C., Lemos L., Reis D.S., Moreira T.G., Souza A.L., Faria A.M.C. (2015). Hydrolyzed whey protein prevents the development of food allergy to β-lactoglobulin in sensitized mice. Cell. Immunol..

[2] Nuomin Baek R., Tsuruta T., Nishino N. (2023). Modulatory Effects of A1 Milk, A2 Milk, Soy, and Egg Proteins on Gut Microbiota and Fermentation. Microorganisms.

[3] Giribaldi M., Lamberti C., Cirrincione S., Giuffrida M.G., Cavallarin L. (2022). A2 milk and BCM-7 peptide as emerging parameters of milk quality. Front. Nutr..

[4] Kaplan M., Baydemir B., Günar B.B., Arslan A., Duman H., Karav S. (2022). Benefits of A2 Milk for Sports Nutrition, Health and Performance. Front. Nutr..

[5] Daniloski D., McCarthy N.A., Huppertz T., Vasiljevic T. (2022). What is the impact of amino acid mutations in the primary structure of caseins on the composition and functionality of milk and dairy products?. Curr. Res. Food Sci..

[6] Daniloski D., McCarthy N.A., Vasiljevic T. (2022). Impact of heating on the properties of A1/A1, A1/A2, and A2/A2 p-casein milk phenotypes. Food Hydrocoll..

[7] Asledottir T., Le T.T., Poulsen N.A., Devold T.G., Larsen L.B., Vegarud G.E. (2018). Release of β-casomorphin-7 from bovine milk of different β-casein variants after ex vivo gastrointestinal digestion. Int. Dairy J..

[8] Semwal R., Joshi S.K., Semwal R.B., Sodhi M., Upadhyaya K., Semwal D.K. (2022). Effects of A1 and A2 variants of β-casein on human health—Is β-casomorphin-7 really a harmful peptide in cow milk?. Nutrire.

[9] Barnett M.P., McNabb W.C., Roy N.C., Woodford K.B., Clarke A.J. (2014). Dietary A1 β-casein affects gastrointestinal transit time, dipeptidyl peptidase-4 activity, and inflammatory status relative to A2 β-casein in Wistar rats. Int. J. Food Sci..

[10] Haq M.R.U., Kapila R., Sharma R., Saliganti V., Kapila S. (2013). Comparative evaluation of cow β-casein variants (A1/A2) consumption on Th2-mediated inflammatory response in mouse gut. Eur. J. Nutr..

[11] Chia J.S., McRae J.L., Enjapoori A.K., Lefèvre C.M., Kukuljan S., Dwyer K.M. (2018). Dietary Cows’ Milk Protein A1 Beta-Casein Increases the Incidence of T1D in NOD Mice. Nutrients.

[12] Sodhi M., Mukesh M., Kataria R.S., Mishra B.P., Joshii B.K. (2012). Milk proteins and human health: A1/A2 milk hypothesis. Indian J. Endocrinol. Metab..

[13] Ng-Kwai-Hang K.F., Grosclaude F. (1994). Genetic Polymorphism of Milk Proteins. Int. Dairy J..

[14] McBean G.J., Aslan M., Griffiths H.R., Torrão R.C. (2015). Thiol redox homeostasis in neurodegenerative disease. Redox Biol..

[15] Murakami M., Nakatani Y., Atsumi G.I., Inoue K., Kudo I. (1997). Regulatory Functions of Phospholipase A2. Crit. Rev. Immunol..

[16] Pan Y., Xie Q.T., Zhu J., Li X.M., Meng R., Zhang B., Jin Z.Y. (2019). Study on the fabrication and in vitro digestion behavior of curcumin-loaded emulsions stabilized by succinylated whey protein hydrolysates. Food Chem..

[17] Zhao Y., Wan Z., Zhang R., Ma X., Wang M., Shao S., QI B. (2020). Optimization of Composition Proportion of Compound Ginseng Immune-enhancing Formula and Study of Its Immunomodulatory Activity and Acute Toxicity on Mice. China Pharm..

[18] Shi J. (2020). Immune-mediatory and Intestinal Barrier Function of Two Glycated Caseinate Digests. Ph.D. Dissertation.

[19] Food and Drug Administration (2005). Guidance for Industry: Estimating the Maximum Safe Starting Dose in Initial Clinical Trials for Therapeutics in Adult Healthy Volunteers.

[20] Hao L.X., Zhao X.H. (2016). Immunomodulatory potentials of the water-soluble yam (Dioscorea opposita Thunb) polysaccharides for the normal and cyclophosphamide-suppressed mice. Food Agric. Immunol..

[21] Delves P.J., Martin S.J., Burton D.R., Roitt I.M. (2011). Roitt’s Essential Immunology.

[22] Albert M.J., Raghupathy R., Khan I., Azizieh F.Y. (2019). In vitro spleen cell cytokine responses of adult mice immunized with a recombinant PorA (major outer membrane protein [MOMP]) from Campylobacter jejuni. Sci. Rep..

[23] Monmai C., You S., Park W.J. (2019). Immune-enhancing effects of anionic macromolecules extracted from Codium fragile on cyclophosphamide-treated mice. PLoS ONE.

[24] Won T.J., Kim M.S., Woo J.S., Han S.B., Hwang K.W. (2007). Hematopoietic Effect of Phellinus linteus Polysaccharide in Mouse Splenocytes and Bone Marrow Cells. Biomol. Ther..

[25] Mercier A., Gauthier S.F., Fliss I. (2004). Immunomodulating effects of whey proteins and their enzymatic digests. Int. Dairy J..

[26] Wang Y., Meng Q., Qiao H., Jiang H., Sun X. (2009). Role of the Spleen in Cyclophosphamide-induced Hematosuppression and Extramedullary Hematopoiesis in Mice. Arch. Med. Res..

[27] Liu Y., Zheng D., Wang D., Su L., Wang Q., Li Y. (2019). Immunomodulatory Activities of Polysaccharides from White Button Mushroom, *Agaricus bisporus* (Agaricomycetes), Fruiting Bodies and Cultured Mycelia in Healthy and Immunosuppressed Mice. Int. J. Med. Mushrooms..

[28] Cui H.Y., Wang C.L., Wang Y.R., Li Z.J., Chen M.H., Li F.J., Sun Y.P. (2015). Pleurotus nebrodensis polysaccharide (PN-S) enhances the immunity of immunosuppressed mice. Chin. J. Nat. Med..

[29] Vitale M., Cantoni C., Pietra G., Mingari M.C., Moretta L. (2014). Effect of tumor cells and tumor microenvironment on NK-cell function. Eur. J. Immunol..

[30] Gordon S., Martinez F.O. (2010). Alternative Activation of Macrophages: Mechanism and Functions. Immunity.

[31] Liu B., Qiao W., Zhang M., Liu Y., Zhao J., Chen L. (2022). Bovine milk with variant β-casein types on immunological mediated intestinal changes and gut health of mice. Front. Nutr..

[32] Guantario B., Giribaldi M., Devirgiliis C., Finamore A., Colombino E., Capucchio M.T., Roselli M. (2020). A Comprehensive Evaluation of the Impact of Bovine Milk Containing Different Beta-Casein Profiles on Gut Health of Ageing Mice. Nutrients.

[33] Parker B.J., Wearsch P.A., Veloo A.C., Rodriguez-Palacios A. (2020). The Genus Alistipes: Gut Bacteria with Emerging Implications to Inflammation, Cancer, and Mental Health. Front. Immunol..

[34] David L.A., Maurice C.F., Carmody R.N., Gootenberg D.B., Button J.E., Wolfe B.E., Turnbaugh P.J. (2014). Diet rapidly and reproducibly alters the human gut microbiome. Nature.

[35] Wanders D., Graff E.C., Judd R.L. (2012). Effects of high fat diet on GPR109A and GPR81 gene expression. Biochem. Biophys. Res. Commun..

[36] Morrison D.J., Preston T. (2016). Formation of short chain fatty acids by the gut microbiota and their impact on human metabolism. Gut Microbes.

[37] Kim M., Qie Y., Park J., Kim C.H. (2016). Gut Microbial Metabolites Fuel Host Antibody Responses. Cell Host Microbe.

[38] Zhang J., Zhou H.C., He S.B., Zhang X.F., Ling Y.H., Li X.Y., Hou D.D. (2021). The Immuno-Enhancement Effects of Sea buckthorn pulp oil in Cyclophosphamide-Induced Immunosuppressed Mice. Food Funct..

